# Weak-formulated physics-informed modeling and optimization for heterogeneous digital materials

**DOI:** 10.1093/pnasnexus/pgae186

**Published:** 2024-05-08

**Authors:** Zhizhou Zhang, Jeong-Ho Lee, Lingfeng Sun, Grace X Gu

**Affiliations:** Department of Mechanical Engineering, University of California, Berkeley, Berkeley, CA 94720, USA; Department of Mechanical Engineering, University of California, Berkeley, Berkeley, CA 94720, USA; Department of Mechanical Engineering, University of California, Berkeley, Berkeley, CA 94720, USA; Department of Mechanical Engineering, University of California, Berkeley, Berkeley, CA 94720, USA

**Keywords:** physics-informed neural networks, neural operators, digital materials, weak formulation, topology optimization

## Abstract

Numerical solutions to partial differential equations (PDEs) are instrumental for material structural design where extensive data screening is needed. However, traditional numerical methods demand significant computational resources, highlighting the need for innovative optimization algorithms to streamline design exploration. Direct gradient-based optimization algorithms, while effective, rely on design initialization and require complex, problem-specific sensitivity derivations. The advent of machine learning offers a promising alternative to handling large parameter spaces. To further mitigate data dependency, researchers have developed physics-informed neural networks (PINNs) to learn directly from PDEs. However, the intrinsic continuity requirement of PINNs restricts their application in structural mechanics problems, especially for composite materials. Our work addresses this discontinuity issue by substituting the PDE residual with a weak formulation in the physics-informed training process. The proposed approach is exemplified in modeling digital materials, which are mathematical representations of complex composites that possess extreme structural discontinuity. This article also introduces an interactive process that integrates physics-informed loss with design objectives, eliminating the need for pretrained surrogate models or analytical sensitivity derivations. The results demonstrate that our approach can preserve the physical accuracy in data-free material surrogate modeling but also accelerates the direct optimization process without model pretraining.

**Significance Statement**

Advancement in physics-informed neural networks (PINNs) allows data-free machine learning of differential equations, paving the way for harnessing the power of machine learning in tackling complex scientific challenges. However, the learning logic of PINNs poses challenges in problems containing highly discontinuous material domains, for instance, 3D-printed digital materials. In this work, a weak-formulated residual loss is introduced to address the discontinuity hurdle of PINNs. The proposed framework can model pixelated composites or digital materials efficiently and accurately. We further propose a physics-informed design optimization workflow by integrating the weak residual loss with design objective functions. This significantly accelerates the optimization process by eliminating model pretraining, marking a potential leap forward in complex scientific design exploration.

## Introduction

Numerical simulations have been the most widely adopted technique in solving partial differential equations (PDEs) to help engineers and researchers understand real-world physics, for instance, analyzing and designing structural materials (1–6). As the most prevailing numerical formulation for structural mechanics, the finite element method (FEM) has allowed researchers and engineers to screen through design candidates much faster compared to physical experiments. Nevertheless, the computational cost of FEM scales at least quadratically with respect to the degrees of freedom in a material mesh. This need for data efficiency exists also from the perspective of the optimization algorithm itself, especially when designing digital materials (7, 8). Classical techniques for material structural optimization can be generally categorized as black box searching and gradient-based optimization algorithms. Black box searching methods such as genetic algorithms, simulated annealing, Bayesian optimization, and cross-entropy among others (9–12) are highly generalizable with straightforward configurability but fail on high-dimensional problems due to low data efficiency. On the other hand, when sensitivity (gradient) information is accessible, the optimization can be accelerated by orders of magnitude given a good initialization, especially for tasks with large design space such as topology optimization (13–16). However, a good design initialization is itself a challenge, and the method requires derivation of first-order or sometimes second-order analytical sensitivities which are problem-specific and difficult to implement, significantly restricting their industrial applications.

In the past decade, machine learning (ML) has been one of the most widely studied research subjects, for its ability to tackle big data in a large parameter space (17–23). Although ML was originally developed to focus on vision and language tasks, it shows potential in exploring large material design space when numerical simulations are too costly. So far, ML is primarily used to train surrogate neural network models based on materials that have been readily labeled by simulations or experiments. Unlike numerical simulations, a surrogate model (24–28) makes inferences at nearly zero cost and can therefore perform design optimization by fast screening through unseen material structures. However, the performance of surrogate model based material optimization oftentimes depends on a rich training dataset, which is oftentimes inaccessible or too expensive for material problems. To reduce data dependency, researchers have proposed physics-informed neural networks (PINN) that learn from governing equations, typically partial differential equations (PDE), rather than ground truth data (29). PINNs have been widely studied to address various engineering physics problems including solid mechanics (30), fluid dynamics (31), electromagnetism (32), etc. The success of PINN owes to the auto-differentiability of neural networks, allowing direct estimation of the differential terms, and thus PDE residuals. However, the application of this physics-informed training flow is difficult when the physical field property is highly discontinuous, for instance, in digital materials.

Digital materials represent a type of composite material design space by treating them as assemblies of versatile base material elements (33). Composites, with their unique ability to integrate the strengths of different materials, offer solutions that single bulk materials cannot achieve. Recent advancements in 3D-printing technology have enabled the precise placement of different microscopic material building blocks at desired locations (34–37). This significantly expands the material design space beyond simple parameterization, demanding composites to be defined as digital materials. Meanwhile, analyzing digital materials through PINN is challenging as the mechanical properties of any two neighbor material elements can show large disparities.

Directly learning the differential equation on digital materials is intractable due to their complex property fields. Therefore, researchers have explored variational formulations for general spatial–temporal problems (38, 39), and energy formulations for static equilibrium problems (40, 41), which are equivalent to the variational form when inertia or body force is absent (8, 42). In this work, we consider both the variational and energy formulations as weak formulations, since they perform spatial integrals on physics quantities instead of requiring spatial differentiation of the material property field. This “weaker” requirement in differentiability allows physics-informed training for highly discontinuous digital materials. Integral for physics-informed loss functions are typically estimated through Monte Carlo (43, 44), a direct sampling and summation method, which is proper for domains with few discontinuities. For digital material, however, the level of discontinuity is essentially the number of element interfaces in a mesh. Therefore, the proposed method instead estimates the weak-formulated loss through Gaussian quadratures (45), which generalizes our previous work (46) to the entire spatial–temporal formulation. As material points always possess finite velocity and acceleration, the inertia term remains accessible through backpropagating the neural network.

Besides the quadrature-based weak-formulated physics-informed training, we further propose a physics-informed topology optimization scheme that does not require a trained surrogate model or deriving analytical sensitivity. There have been numerous research works using the power of neural networks as parameterization tools for the physical field (47, 48) or design space (49, 50) of topology optimization problems. However, previous works rely on prior domain knowledge with significant expertise, specifically a derived sensitivity analysis. For practical nonlinear problems, sensitivity analysis requires the adjoint field method (51) which oftentimes requires complex derivation or is even intractable depending on engineering constraints. To alleviate the complex derivation process and accelerate engineering design cycles, we take advantage of the physics-informed training and convert a typical PDE-constrained single-objective topology optimization problem into a multiobjective optimization problem. This is achieved by performing concurrent optimization and physics-informed training on a neural network over the joint space of the spatial domain input and the design parameters. The entire learning process iteratively estimates sensitivity through the semi-trained neural network while correcting the network parameters to minimize the residual loss.

## Results and discussion

In this study, we seek to formulate PINNs for digital materials which possess strong discontinuity. We propose adopting the weak-formulated PDE residual with quadrature integrals (detailed residual forms in the Methods section) to represent the physics loss in digital material problems. The weak-formulated residual is first examined on a dynamic problem where the governing equation takes the most general form on a spatial–temporal domain. The inertia force plays a critical role in this problem as time derivatives can still be computed through auto gradient (unlike the spatial formulation). Therefore, the performance of the weak-formulated residual is also examined on a static equilibrium problem where the inertia force term is removed from the governing equation, facilitating the energy loss that involves no auto gradient. The proposed weak-formulated PINN is then applied to solve dynamic and static equilibrium digital material problems through purely self-supervised training. Furthermore, we will demonstrate how such physics-informed prior can be incorporated into design optimization of digital materials, so that neither the sensitivity analysis nor a pre-trained surrogate model is needed. It is of note here that for simplicity, all physical quantities discussed in this work are dimensionless.

### Prediction for dynamic problems

**Fig. 1.**
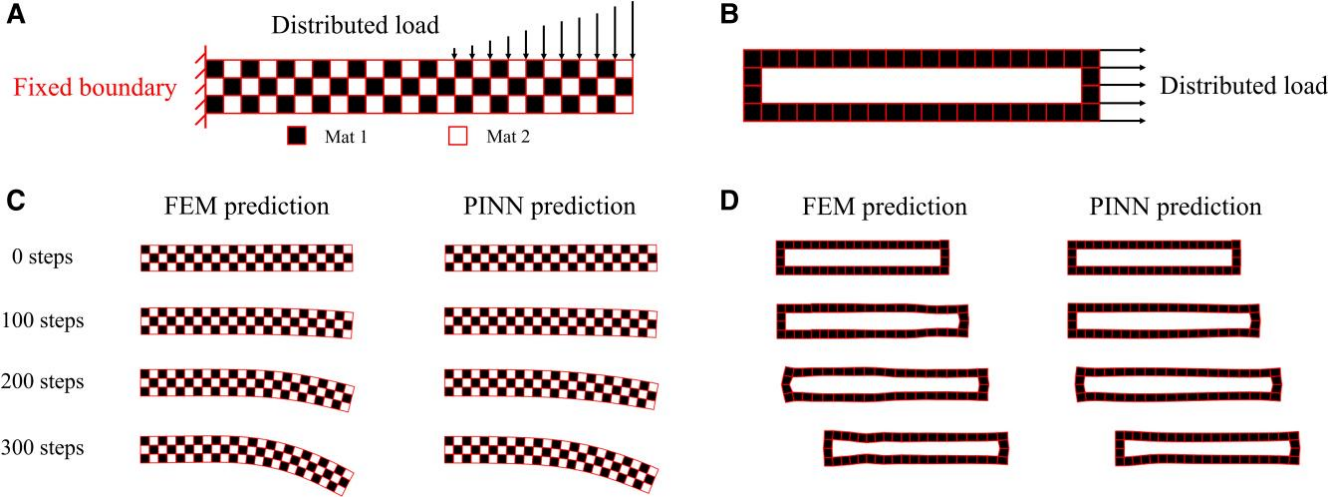
A) Geometry configuration of a dynamic composite beam bending problem, B) geometry configuration of a dynamic hollow block acceleration problem, C) bending deformation of the heterogeneous composite beam at 0, 100, 200, and 300 time steps. The left column shows results from FEM and the right column shows results from the weak-formulated PINN, D) acceleration and deformation of the hollow block at 0, 100, 200, and 300 time steps. The left column shows results from FEM and the right column shows results from the weak-formulated PINN.

We first examine the capability of the proposed weak-formulated PINN as a forward solver to dynamic digital material deformation problems. Figure 1A shows the general configuration of the first benchmark problem: the bending process of a composite beam subject to a distributed load pressing on the tip. The beam domain *Ω* has a length of 24 and width of 3, which is meshed into 72 rectangular elements. The mesh serves not only as the numerical discretization but also the digital material domain representation. The mesh includes 100 nodes denoted as $\Omega^{d}$, and 4 fixed boundary nodes denoted as $\partial \Omega^{b}\subset \partial \Omega^{d}\subset \partial \Omega$. It is subject to a zero displacement $u^{i}(X,t=0)=0$ and velocity $\frac{\partial u^{i}}{\partial t}(X,t=0)=0$ as the initial condition for all $X\in \Omega^{d}$. Here, X, *t*, and $u^{i}$ denote the material coordinate, time, and the prescribed initial displacement function. A Dirichlet boundary condition is applied to the left beam end with $u^{b}(X,t)=0$ for all $(X,t)\in \partial \Omega^{b}\times T$. Here, $u^{b}$ denotes the prescribed boundary displacement function. The boundary and initial conditions can be written as training loss:


(1) $$\begin{array}{c} {bond}_{m}=NN_{\theta }(X_{m},t_{m})-u^{b}(X_{m},t_{m}),\\ \;\forall (X_{m},t_{m})\in \partial \Omega^{b}\times T, \end{array}$$

(2) $$\begin{array}{c} {initu}_{n}=NN_{\theta }(X_{n},t=0)-u^{i}(X_{n},t=0),\\ \;\forall X_{n}\in \Omega^{d}, \end{array}$$

(3) $$\begin{array}{c} {initv}_{q}=\frac{\partial NN_{\theta }}{\partial t}(X_{q},t=0)-\frac{\partial u^{i}}{\partial t}(X_{q},t=0),\\ \;\forall X_{q}\in \Omega^{d}. \end{array}$$

A ramped load distribution (scaled linearly from p=0 to p=0.3 pointing downward) is applied to the 11 top right boundary nodes of the composite beam, as highlighted by the arrows in Fig. 1A. We assign the maximum extent of heterogeneity to the composite beam by alternating the material between each pair of neighboring elements. The black elements possess a modulus of 90 and a density of 10, which are halved for the white elements. The entire composite beam shares a constant Poisson’s ratio of 0.3. A neural network $NN_{\theta }(X,t)$ is initialized to solve for the deformation behavior of the composite beam on a set of time points T⊂[0,2]. 300 equally spaced time points are sampled as *T*, yielding a total number of 30,000 spatial–temporal collocation points. The complete training loss is then expressed in Eq. 4 below where the PDE residual r is calculated from Eq. 14, and the absolute value signs indicate the size of the sets. The self-adaptive training method is adopted here, which assigns the adaptive weights *α*, *β*, *γ*, and *η* in front of all the loss terms. The adaptive weights are updated to maximize the loss while the neural network weights *θ* are updated to minimize the loss, which is proved to significantly help overcome local minimums during training (52):


(4) $$\begin{array}{rl} L & =\min_{\theta }\max_{\alpha ,\beta ,\gamma ,\eta }\frac{1}{\left|\partial \Omega^{b}\times T\right|}\sum_{m}\alpha_{m}{bond}_{m}^{2}\\ & \;+\frac{1}{\left|\Omega^{d}\right|}\sum_{n}\beta_{n}{initu}_{n}^{2}+\frac{1}{\left|\Omega^{d}\right|}\sum_{q}\gamma_{q}{initv}_{q}^{2}\\ & \;+\frac{1}{\left|\Omega^{d}\times T\right|}\sum_{k}\eta_{k}r_{k}^{2}. \end{array}$$

Figure 1B shows the general configuration of the second benchmark problem: the acceleration process of a soft hollow material block subject to a distributed pulling load on the right end. The hollow block has a length of 20, width of 4, and wall thickness of 1, with its center being hollow, which is meshed into 44 rectangular elements. The mesh includes 44 elements and 88 nodes for $\Omega^{d}$. It is subject to a zero displacement $u^{i}(X,t=0)=0$ and velocity $\frac{\partial u^{i}}{\partial t}(X,t=0)=0$ as the initial condition for all $X\in \Omega^{d}$. No Dirichlet boundary condition is applied in this problem and the material block is allowed to move freely. A uniformly distributed load with a magnitude of 10 is applied on the right end, pulling the entire hollow block to deform and accelerate. This pulling force manifests as the external traction p=2 on each of the five boundary nodes as highlighted in Fig. 1B. Although the material block is homogeneous itself, it can be treated as a heterogeneous body with extremely soft and light material at the center. The black material elements possess a modulus of 90 and a density of 10, with a Poisson’s ratio of 0.3. A neural network $NN_{\theta }(X,t)$ is initialized to solve for the deformation behavior of the composite beam on a set of time points T⊂[0,2]. 300 equally spaced time points are sampled as *T*, yielding a total number of 26,400 spatial–temporal collocation points. In both the composite beam and hollow body scenarios, the learning rate is initialized to 0.005 and is scaled by 0.9 for every 200 training epochs.

Figure 1C shows the simulation results for the composite beam bending scenario from the classical FEM solver, and our weak-formulated PINN, plotted at $t=\frac{2}{3}$, $\frac{4}{3}$, and 2 (i.e. 100, 200, and 300 time steps). The prediction from the weak-formulated PINN matches well with the FEM results in the general deformation trend. However, it can be observed that the weak-formulated PINN tends to bend the entire composite beam as a whole compared to the FEM prediction where the loaded beam tip portion shows a more notable deformation. On the other hand, Fig. 1D demonstrates the simulation results for the hollow block pulling scenario from the classical FEM solver, and our weak-formulated PINN, plotted at $t=\frac{2}{3}$, $\frac{4}{3}$, and 2. In addition, the initial state at t=0 is also shown on the top to better visualize the displacement of the entire material block. From the computation results of both methods, we can observe that the distributed pulling load on the right tip starts by extending the block body. The elongated material block then contracts and accelerates toward the pulling direction. Meanwhile, this extension–contraction cycle also generates a transverse elastic wave along the hollow block, causing wavy deformation on the block wall. The weak-formulated PINN captures most macroscopic behaviors of the hollow block including acceleration and the extension–contraction cycle. However, a clear difference can be observed for the microscopic wave- propagation on the block wall, which is instead treated by the weak-formulated PINN as a wave with a larger wavelength but smaller magnitude.

**Fig. 2.**
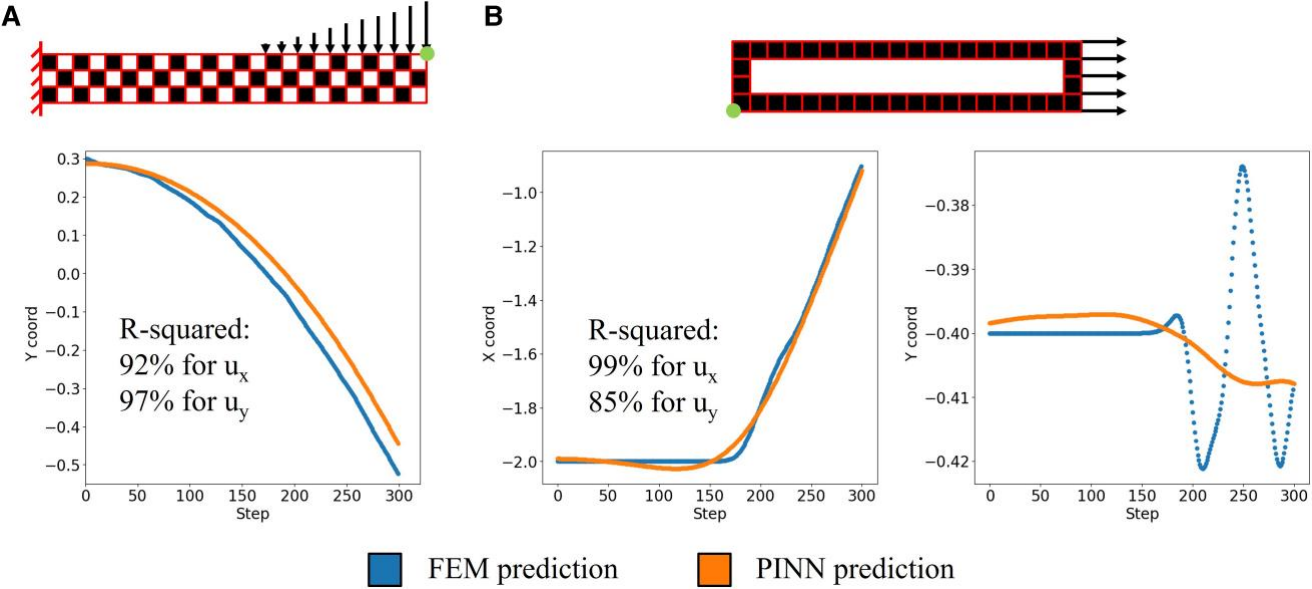
A) The leftmost plot shows the vertical displacement of the top right node highlighted by the circular (green) dot for the composite beam bending problem. The PINN results are compared to FEM, showing good agreement. B) The middle and right plots show the horizontal and vertical displacement of the bottom left node highlighted by the circular (green) dot for the hollow block pulling problem. The PINN results are compared to FEM. A clear difference can be observed in the vertical displacement plot.

To better quantify the performance of the weak-formulated PINN, we plot the displacement evolution of the most characteristic node in the two test problems as shown in Fig. 2. In both problems, the weak-formulated PINN accurately captures the macroscopic movement of the highlighted nodes, such as the horizontal and vertical displacements of the beam tip node, and the horizontal displacement of the block corner node. The R-squared values calculated over the spatiotemporal input space (expressed as Eq. 5) achieve more than 90% for macroscopic displacement predictions:


(5) $$R^{2}(u(X,t),\hat{u}(X,t))=\frac{\sum_{(X,t)\in \Omega^{d}\times T}(u(X,t)-\hat{u}(X,t){)}^{2}}{\sum_{(X,t)\in \Omega^{d}\times T}(\hat{u}(X,t)-mean(\hat{u}){)}^{2}},$$

where mean(u) represents the averaged nodal displacement values from FEM. On the contrary, the microscopic nodal movement, for instance, the vertical displacement of the material block corner node (a good indicator for the wave deformation on the block wall), deviates from the FEM solution. The averaged R-squared value for horizontal displacement prediction of the hollow block nodes is 85%, lower than the macroscopic predictions.

As a forward solver, weak-formulated PINN successfully overcomes the extreme discontinuity of digital materials and captures general dynamic behaviors with no need for ground truth observations. However, the spatial quadrature integrals tend to stiffen the trainable parameters, causing the model to prefer smaller time derivatives. In Fig. 2A, this stiffening effect manifests as a more uniform deformation over the entire composite beam. While in Fig. 2B, an elastic wave can be observed in the FEM result, caused by the sudden acceleration of the soft thin wall material structure (also manifest in Fig. 1D). This vibration has a relatively small magnitude compared to the horizontal motion of the hollow block (Fig. 2B). Therefore, the PINN collapses at a solution that averages out the vibration which is a local minimum of the spatial–temporal residual due to the stiffening effect. As the weak-formulated PINN is showing inferior performance compared to numerical solvers on dynamic problems, it’s rational to expect similar outcomes when used as surrogate models when the entire joint space of design parameters and spatial–temporal computation domain is considered. Furthermore, the training time (approximately 5 min) is longer than our FEM model based in Matlab (5 s) for this dynamic problem. However, when the inertia term is absent, weak-formulated PINN shows significantly better potential when performing modeling and optimization tasks for static equilibrium problems, as shown in the following sections.

### Surrogate modeling for static equilibrium problems

**Fig. 3.**
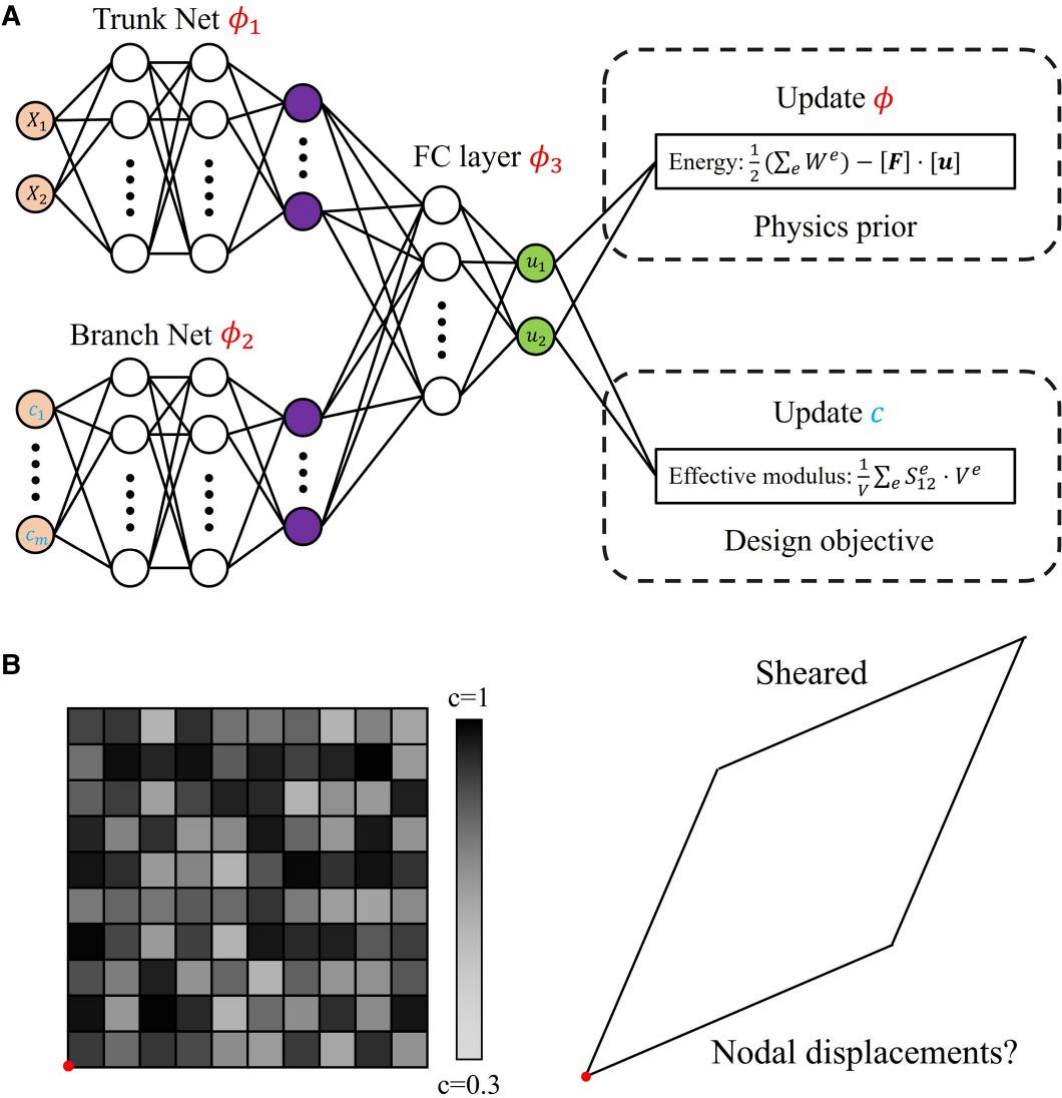
A) Schematic showing the surrogate model architecture and loss functions. The model adopts the DeepONet architecture with a trunk net, a branch net, and some concatenated layers. Proper deformation predictions are obtained through energy minimization while design optimization can be achieved through objective maximization, B) A 2D digital material (10×10) shear problem with the bottom left node fixed. The entire 2D sheet is experiencing a macroscopic shear strain of 0.2. Each material voxel is assigned a density value (design parameter) between c=0.3 and c=1.

Besides dynamic responses, we have also studied the performance of the proposed weak formulation PINN in addressing static equilibrium problems, indicating a zero acceleration $a=\frac{\partial^{2}u}{\partial t^{2}}=0$. In this case, Eq. 14 can be simplified to r=[R]−[F]. Alternatively, we adopt the minimum energy principle (8) which states that the solution to Eq. 14 is equivalent to minimizing the system energy *U* shown below under static equilibrium scenarios:


(6) $$U=\frac{1}{2}\sum_{e}W^{e}-[F]\cdot [u]=\frac{1}{2}\sum_{e}\int_{\Omega^{e}}S^{e}\;:\;E^{e}d\Omega -[F]\cdot [u],$$

where the *e* superscript is the index for mesh element, [u] is the assembled displacement vector for all nodes, and $W^{e}$ denotes the strain energy of element *e*. Instead of a mere equation solver, we complicate the task by letting the neural network learn a surrogate model $NN_{\theta }(X,c)\;:\;\Omega \times R^{m}\to R^{2}$ that predicts the displacement vector u at a material coordinate X given the digital material design representation c of length *m* depending on the number of adjustable material elements. The time argument is removed as the model only predicts static equilibrium solutions. We use a DeepONet architecture (53) for the surrogate model as illustrated in Fig. 3A, containing a branch net, and a trunk net. The trunk net has an input size of 2 and 3 hidden layers of size 50. The branch net has an input size of *m* and 3 hidden layers of size 128. Instead of the dot product (based on linear dependency assumption), we choose to concatenate the outputs from branch net and trunk net, which then passes through another 2 hidden layers of size 100. All activation functions are using the hyperbolic tangent.

The surrogate model parameters *ϕ* will be trained solely in a physics-informed manner, through the minimization of system energy given by Eq. 6. The integral for strain energy *W* is calculated elementwise through Gaussian quadrature as discussed in the previous section. In addition, the material design parameters c are set to be trainable by an objective function which will be discussed in the subsequent Design optimization section. Figure 3B shows the configuration of the static equilibrium problem that the surrogate model attempts to learn. The problem contains a 2D digital material experiencing a prescribed macroscopic shear strain, directly applied as Dirichlet boundary conditions on all of the four edges. The entire material domain is discretized into 10×10 elements each with a size of 0.2×0.2. This yields a total of 121 nodes as collocation points ($|\Omega^{d}|=121$) with 40 of them being constrained on boundaries. As there are no external loadings in this problem, the work term [F]⋅[u] can be omitted from energy calculation (Eq. 6). The material elements obey isotropic linear elasticity where Young’s Modulus stays in the range of 6−50, and the Poisson’s ratio is fixed at 1/3. As the geometry does not possess any symmetry in this case, the feature vector c represents the normalized Young’s Modulus with a length of m=100.

A total of 20,000 random material configurations are sampled as input features for training. Obtaining the ground truth label using FEM for each data point would take approximately 0.5 s if the model is trained in a supervised manner. Unlike residual minimization, self-adaptive weights are unnecessary for the scalar energy loss. Moreover, Dirichlet boundary conditions can be applied simply and exactly. Specifically, the neural network predictions at boundary nodes are masked and replaced with prescribed displacement values, while predictions at interior nodes remain unchanged. The residual is then calculated based on the masked and updated predictions. The material system energy is therefore minimized for 150 epochs with a batch size of 40. The initial learning rates for all trainable parameters are set to 0.005 with exponential scheduling scaled by 0.97 for every 3 epochs. The hyperparameters are tuned by observing the descent of system energy from Eq. 6 without the need for a ground-truth validation set. Another 2,000 sets of features are randomly generated and labeled by FEM simulations for testing. The energy calculation of material energy is parallelized over all batch data, mesh elements, and quadrature points to accelerate the training process. The entire training process takes approximately 60 min to accomplish, and the trained PINN surrogate model shows an R-squared value (Eq. 7) of 99% for the nodal displacement predictions on the test data set, where boundary nodes are excluded from accuracy evaluation as they are directly masked:


(7) $$\begin{array}{rl} & R^{2}(u(X,c),\hat{u}(X,c))\\ & \;=\frac{\sum_{(X,c)\in (\Omega^{d}\setminus \partial \Omega^{d})\times R^{m}}(u(X,c)-\hat{u}(X,c){)}^{2}}{\sum_{(X,c)\in (\Omega^{d}\setminus \partial \Omega^{d})\times R^{m}}(\hat{u}(X,c)-mean(\hat{u}){)}^{2}}. \end{array}$$

**Fig. 4.**
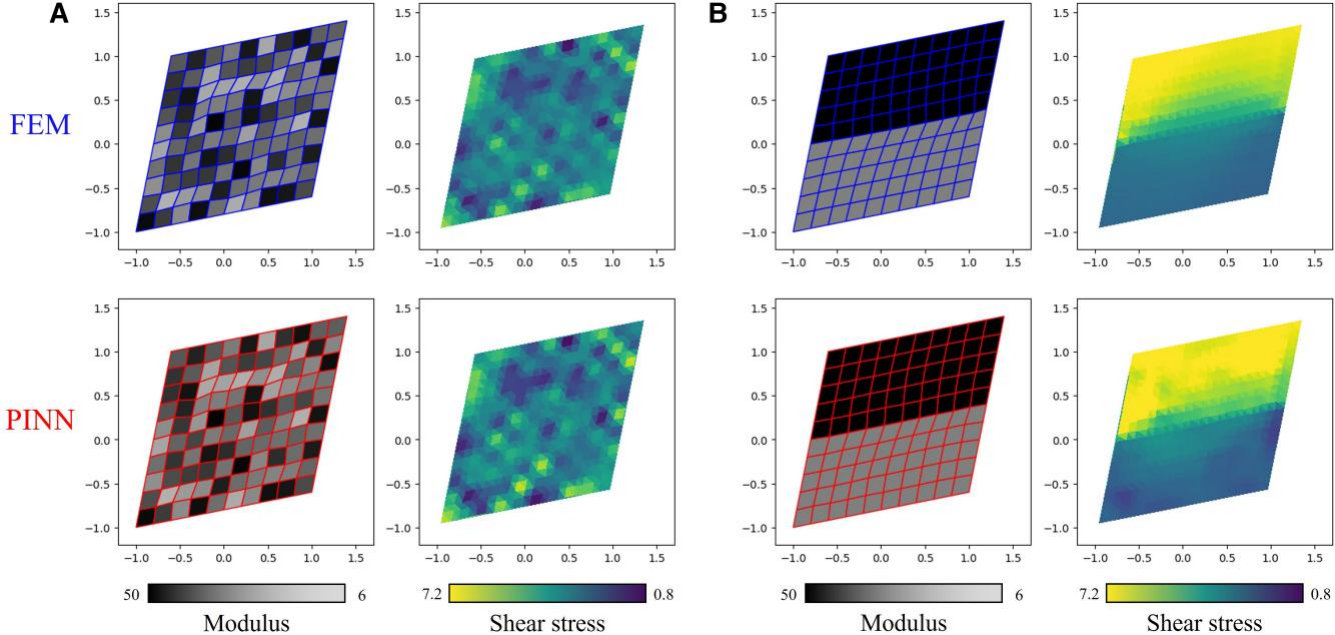
Comparison of the mesh deformation and resultant shear component of the second Piola–Kirchoff stress S between the FEM simulations and the PINN surrogate model predictions. Results are demonstrated for A) a digital material with randomly assigned element properties and B) a manually configured digital material with a modulus of 50 on the top half, and a modulus of 6 on the bottom.

Figure 4A shows the prediction of the trained neural network $NN_{\theta }$ with a randomly chosen material distribution (beyond the training set) for the static equilibrium shear problem. The predictions are compared with FEM results using the classical Newton–Raphson solver. The nodal displacement field as well as the shear stress (evaluated at the quadrature points) agrees well with numerical simulation results. Figure 4B shows a manually configured material distribution with the top half sheet being stiff (modulus of 50) while the bottom half is soft (modulus of 18.5). This configuration is specifically chosen to examine the model’s performance in extreme cases, which differ significantly from training data that have scattered material configurations as in Fig. 4A. From both the FEM and the surrogate model predictions, distortion can be observed on the midline of the digital material sheet where the shear stress stays continuous despite the sharp material property transition. We notice that the shear stress predictions tend to exhibit greater differences than displacement field predictions when compared to FEM results. The FEM plot shows a smooth stress transition towards the material boundary as a result of the zero stress divergence term in Eq. 12 (no body or inertia force). On the other hand, the PINN prediction shows the correct trend in general stress distribution, but with minor stress differences on smaller scales, specifically the singular stress concentration regions. Such error can be attributed to two major reasons: First, the neural network is trained to produce zero residual in expectation rather than in an exact sense. Second, the stress prediction is derived from the first derivative of the displacement field (output of the model), potentially magnifying minor errors originating from the neural network predictions.

In this section, we have shown the possibility of training surrogate models for digital materials with spatially discontinuous features by following the minimum energy principle derived from the weak formulation. The proposed framework is tested on a static equilibrium shear stretching problem, showing remarkable accuracy and efficiency. It is worth noticing that the removal of the auto gradient inertia term greatly relieves the stiffening effect observed in the dynamic problem, allowing a smoother learning process on the static equilibrium problem. We believe the surrogate model can be further enhanced, by generating new random input features over each training epoch. This allows the model to explore the feature space more thoroughly with no extra computational cost under the physics-informed training framework. Additionally, physics-informed training transforms the PDE-solving process into an optimization challenge. We will demonstrate how this advantage facilitates the design of digital materials without the need for sensitivity analysis or training a full-fledged surrogate model.

### Physics-informed topology optimization

Classical topology optimization methods treat the governing PDE as an equality constraint (54). Utilizing the minimum energy principle, we have shown that the equality constraint can be retreated as a loss function, which can be further combined with an objective function to formulate the physics-informed topology optimization scheme for digital materials. The proposed optimization scheme will be showcased on the static equilibrium shear problem as discussed in Fig. 3B. Specifically, we are interested in finding the optimal material distribution c that maximizes the effective shear modulus of the digital material, defined as the volumetric average of the shear component of the second Piola–Kirchoff stress. This optimization problem can be expressed as Eq. 8 shown below:


(8) $$\left\{ \begin{array}{cc} \max_{c} & \left(G_{\mathrm{eff}}=\frac{1}{V}\sum_{e}\int_{\Omega_{e}}S_{12}^{e}d\Omega \right)\;\\ \text{s.t.} & u={\text{arg min}}_{u}\frac{1}{2}(\sum_{e}\int_{\Omega_{e}}S^{e}\;:\;E^{e}d\Omega )-[F]\cdot [u],\\ & u(X)=G_{\mathrm{mac}}(X-X^{\mathrm{fixed}})\;\forall \;X\in \partial \Omega^{b}\\ & \frac{1}{m}\sum_{e=1}^{m}c_{e}=0.6\;\\ & 0.3\leq c_{e}\leq 1, \end{array}\right.$$

where $G_{\mathrm{mac}}$ denotes the macroscopic shear strain of 0.2, and $X^{\mathrm{fixed}}$ is the fixed red point highlighted in Fig. 3B. Note that the element index *e* is used as a subscript for the design vector c to indicate the *e*th component, but as a superscript in stress and strain tensors to indicate their correspondent element shape function. Taking manufacturability into account, the optimized composite design would ideally consist of only two base material voxels: a soft one with Young’s modulus of 6, and a stiff one with Young’s modulus of 50. To avoid the combinatorial optimization problem from a discrete design space, Young’s modulus *E* of a material element *e* is defined to be a smooth function of the design parameter $E^{e}=6+128\cdot (c_{e}-0.3{)}^{3}$. The design parameters are constrained within a range of [0.3,1] as in Eq. 8 so that the resultant Young’s modulus and shear modulus stay within ranges of [6,50] and [2.25,18.75]. The exponential number of 3 ensures the convexity of the density-modulus projection, encouraging c to converge to their boundary values, which is a widely used technique in topology optimization (55).

A neural network $NN_{\phi }$ is initialized using the DeepONet architecture, with both the weight parameters *ϕ* and design parameters c set to be trainable as shown in Fig. 3A. The neural network does not serve the purpose of a surrogate model as the training process would be cost-inefficient. Instead, the model interpolates among any historical design candidates through the weak-formulated energy loss to help infer design sensitivities. The optimization problem defined in Eq. 8 can be rewritten into two loss functions for training *ϕ* and c, where the first one is the material energy loss as expressed in Eq. 6, and the second one is shown in Eq. 11 below:


(9) $$L_{\mathrm{vol}}=\sum_{e=1}^{m}c_{e}-0.6m,$$

(10) $$L_{\;\mathrm{pos},e}=\sum_{e=1}^{m}ReLU(c_{e}-1)+ReLU(0.3-c_{e}),$$

(11) $$L_{\mathrm{op}}=-G_{\mathrm{eff}}+w_{1}L_{\mathrm{vol}}^{2}+w_{2}L_{\;\mathrm{pos},e}^{2},$$

where the subscript *e* denotes the *e*th component (equivalent to element *e*) of the design vector c, and the weight parameters $w_{1}$ and $w_{2}$ are both set to 2. *ReLU* functions are applied in Eq. 10 to constrain the range of c. The two loss functions (Eq. 6 and 11) cannot be directly combined as the purpose of minimum energy is to produce accurate physical field prediction instead of material design. One thing to notice is that $NN_{\phi }$ only needs to make accurate predictions on the neighborhood of query design vectors c rather than the entire design space, which could save a significant amount of computational cost.

The pseudocode (depicted in Algorithm 1) shows the general implementation of the proposed physics-informed topology optimization scheme for digital materials. The algorithm starts by initializing a batch of random initial designs and continues to execute two loops iteratively. The batch size is set to b=20 which introduces negligible extra cost under GPU parallel computing, but helps overcome local minimums. The first loop updates the DeepONet weight parameters *ϕ* to minimize the total energy of the candidate design batch. The purpose of this loop is to improve prediction accuracy for the current design batch as well as their neighborhood. The network parameters will only be updated for p=100 loops before convergence as we only need the model to infer a rough optimization direction for temporary design candidates. From the semi-trained neural network $NN_{\phi }(X,c)$, we can compute sensitivity for a design candidate as $\frac{\partial L_{\mathrm{op}}}{\partial c_{e}}=\frac{\partial L_{\mathrm{op}}}{\partial u}\frac{\partial u}{\partial c_{e}}$ with respect to the design parameters through backpropagation. The candidate design batch is then updated by an Adam optimizer using the sensitivities while keeping the network parameters fixed. Updates on c are only executed for q=20 loops and remain unconverged before switching to the first loop, as predictions on physical fields are yet inaccurate. Both inner loops start with a learning rate of 0.005 which is scaled by a factor of 0.97 for every five epochs (outer loop). The outer training loop can be terminated after a fixed number of iterations, or when both loss functions are converged.

**Algorithm 1:** Digital material design optimization with physics priors

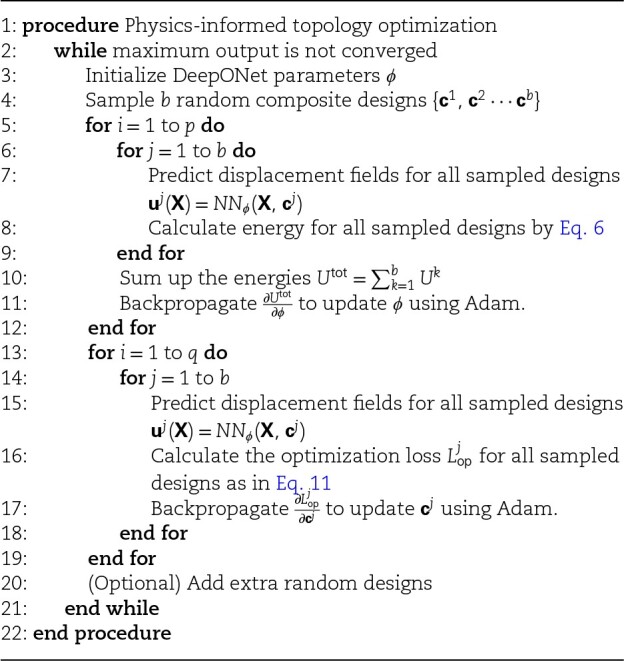

**Fig. 5.**
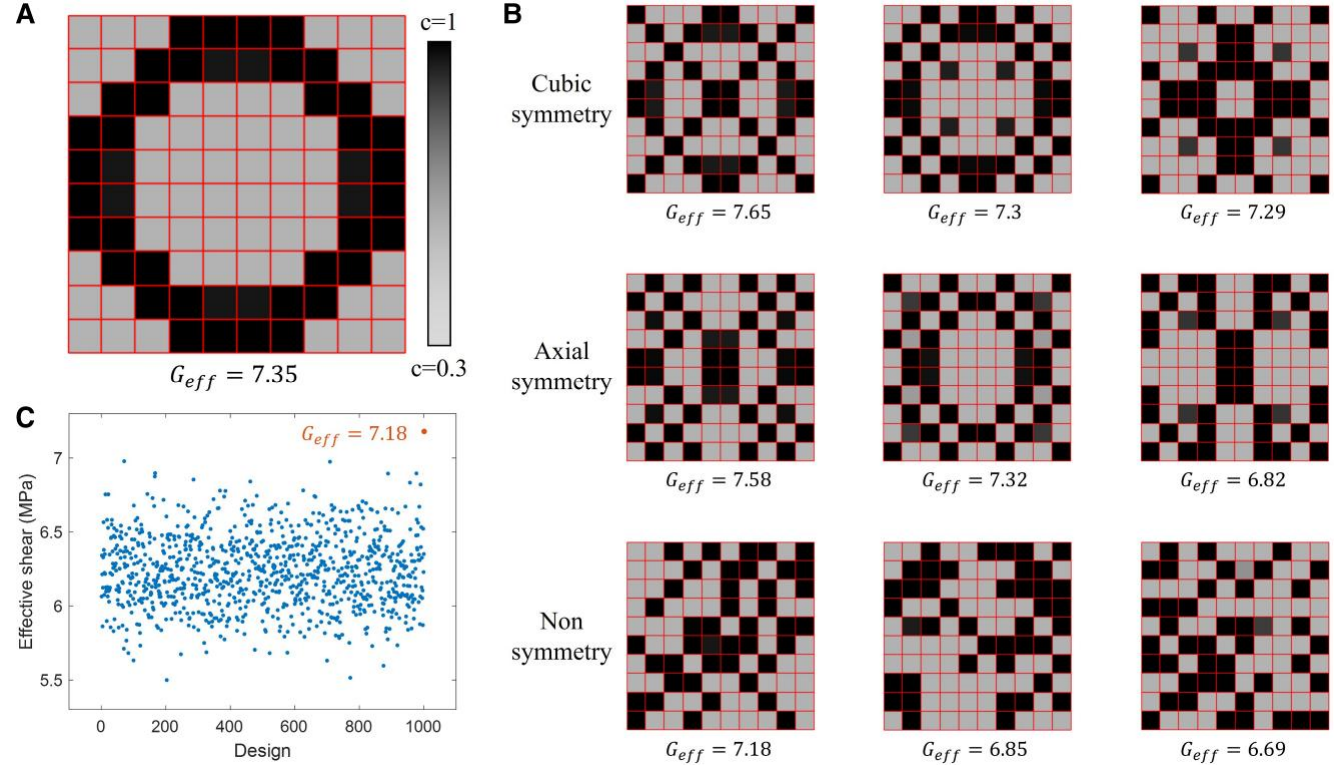
A) The optimized digital material through classical FEM-based SIMP method under the cubic symmetry constraint, B) Some optimized digital material configurations from physics-informed topology optimization when subject to cubic symmetry, axial symmetry, and no symmetry, C) Comparison between the physics-informed optimization result and 1,000 randomly sampled designs without symmetry constraint.

A baseline shear modulus can be estimated by assuming the entire material sheet to be homogeneous at the constrained design parameter (all components of c equal to 0.6), yielding an effective shear modulus of $G_{\mathrm{eff}}=3.55$. By properly arranging the placement of the soft and stiff material elements, we expect a significant improvement from the baseline homogeneous effective shear modulus. For a baseline comparison, we first attempt to optimize the digital material using the classical FEM-based Solid Isotropic Material with Penalisation (SIMP) method implemented in Matlab (56) under the exact same problem definition. The result of SIMP heavily depends on the initialization as it’s not capable of overcoming a local minimal. Figure 5A shows the best SIMP optimized digital material structure out of 200 random initializations, subject to a cubic symmetry constraint. The entire screening process takes approximately 400 s to accomplish. It can be observed that SIMP organizes all stiff material elements into a ring shape to resist shear deformation, with an effective shear modulus of $G_{\mathrm{eff}}=7.35$.

Figure 5B shows some of the optimized designs using our proposed physics-informed topology optimization, when subject to cubic symmetry, axial symmetry, and no symmetry. All the effective shear modulus demonstrated are validated by FEM. When cubic symmetry is imposed, there remains a total of m=15 independent design parameters and the physics-informed topology optimization training process converges in less than 5 min. One candidate shows an effective shear modulus of $G_{\mathrm{eff}}=7.65$ which is slightly better than the results from SIMP. The other two cases also demonstrate outstanding effective shear modulus, that are close to the SIMP result and significantly better than the baseline modulus $G_{\mathrm{eff}}=3.55$. When axial symmetry is applied, there remains a total of m=25 and the training process still converges in less than 5 min. The expanded design space complicates the optimization problem, causing some of the candidate designs trapped at effective modulus values lower than 7. Nevertheless, some candidates managed to find better sub-optimal among which the best design shows superior performance ($G_{\mathrm{eff}}=7.58$) compared to SIMP. When symmetry constraints are completely removed, the full design space with m=100 is searched by the physics-informed topology optimization scheme, taking approximately 10 min to converge. The best design candidate achieves $G_{\mathrm{eff}}=7.18$ which is slightly lower than the SIMP result, while most remaining candidates fall within the range of $G_{\mathrm{eff}}\in [6.6,6.9]$. To better visualize the result, the effective shear modulus of 1,000 randomly generated digital materials with 57 soft elements at $c_{e}=0.3$ and 43 stiff elements at $c_{e}=1$ are plotted in Fig. 5C. It can be observed that the effective shear of the majority of the designs concentrates within the range of $G_{\mathrm{eff}}\in [6,6.5]$, with a minor portion above $G_{\mathrm{eff}}=6.5$. None of the 1,000 random designs get above $G_{\mathrm{eff}}=7$. Despite the large 100 dimensional design space, the proposed algorithm is still capable of finding sub-optimal designs as highlighted by the orange dot in Fig. 5C. It is worth noting that the entire optimization process only requires the weak-formulated residual loss, without a need for further sensitivity derivation. This is realized by the neural operator architecture and direct sensitivity estimation through backpropagation. In other words, instead of a PDE constraint, the governing equation is treated as a second objective function of the optimization problem, allowing automatic computation of sensitivity. Furthermore, such concurrent optimization and learning loops are only available through physics-informed training as data points can be sampled arbitrarily over the entire design space.

## Conclusion

So far, we have discussed the use of a weak-form physics-informed loss in data-free modeling and design optimization of digital materials whose spatial discontinuity is challenging to the prevailing strong-form PINN. The proposed framework achieves high accuracy on static equilibrium problems. However, the quadrature integral tends to stiffen the auto gradients (with respect to the time argument) when inertial terms are introduced from dynamic problems, causing the model to prefer motions with less vibration. The physics-informed training process is more efficient when establishing surrogate models for digital materials, compared to supervised approaches where a huge amount of ground truth data are needed from FEM. On the contrary, it’s not outstanding as a direct PDE solver, as classical numerical methods are faster and more accurate on a single material system. Most importantly, the physics prior can further be incorporated with design objective functions to realize physics-informed topology optimization for digital materials. This optimization framework possesses the following merits: no need for analytical sensitivity analysis; flexible objectives and constraints through adjustment of loss functions; less dependency on design initializations; versatile design outcomes at different local minimals; and better efficiency compared to surrogate model based optimization. When applied to complex and high-dimensional design problems, we believe that surrogate model based optimization methods are costly, but require the least amount of domain knowledge, while the above-mentioned merits prove the potential of our proposed physics-informed optimization. On the other hand, classical methods such as SIMP that rely on sensitivity analysis are the best options for design fine-tuning but can be difficult to implement in practice.

## Methods

### Governing equations

As a solid mechanics problem, the formulation of weak-formulated PINN for digital materials starts by considering the strong-form balance of linear momentum, a material constitutive model, and a strain measure (Eq. 12) as its governing equation:


(12) $$\left\{ \begin{array}{l} \nabla \cdot \sigma +\rho b=\rho a\\ \sigma =\hat{\sigma }(E,\mathrm{Parameters})\\ E=\hat{E}(F)=\hat{E}(I+\frac{\partial u}{\partial X}) \end{array}\right.,$$

where σ, E, *ρ*, b, a, u, and F denote the Cauchy stress tensor, strain tensor, material mass density, body force, acceleration, displacement, and deformation gradient. $\hat{\sigma }$ and $\hat{E}$ are the function form of stress and strain tensors. We adopt the Saint Venant–Kirchhoff constitutive model S=λtr(E)I+2μE where S denotes the second Piola–Kirchoff stress (a pull-back of the Cauchy stress $S=JF^{-1}\sigma F^{-T}$), *λ* and *μ* denote lame constants. Notice that the material is subject to a finite deformation so that the difference between the Piola–Kirchoff stress and Cauchy stress is not negligible. As a result, the nonlinear Green–Lagrangian strain $E=\frac{1}{2}(F^{T}F-I)$ is utilized to measure finite deformation.

### Residual formulation

A neural network $NN_{\theta }(X,t)\;:\;\Omega \times R\to R^{2}$ is initialized to approximate the displacement field u as a function of a material point from the continuum body X∈Ω, and time *t*. Directly evaluating the residual of Eq. 12 requires the computation of $\frac{\partial \lambda }{\partial x}$ and $\frac{\partial \mu }{\partial x}$ to estimate stress divergence (x=X+u denotes the current coordinate), which are not tractable on any of the material transition interfaces. Therefore, we propose to adopt the variational (weak) formulation to calculate the residual r on the reference configuration *Ω* as below (57):


(13) $$r=\int_{\Omega }\xi \cdot \rho_{0}adV+\int_{\Omega }\frac{\partial \xi }{\partial X}\cdot PdV-\int_{\Omega }\xi \cdot \rho_{0}bdV+\int_{\partial \Omega }\xi \cdot pdA,$$

where ξ and $\rho_{0}$ denote the arbitrary test function and material density on the reference configuration. p is the traction force on the undeformed reference configuration, and $P=FS=\frac{\partial x}{\partial X}S$ is the first Piola–Kirchhoff stress.

Digital materials typically use mesh to represent both their material distribution (in elements) and geometries (on nodes). Therefore, given an initialized neural network $NN_{\theta }$, our goal is to optimize its parameters *θ* to minimize the squared residual $r^{2}$ given a set of nodes $X\in \Omega^{d}$ from the material mesh. Time derivatives can be directly obtained from backpropagating the neural network to estimate the acceleration $a(X,t)=\frac{\partial^{2}u(X,t)}{\partial t^{2}}$. Equation 14 shows the numerically approximated form of Eq. 13. The arbitrary test function ξ is factored out as the residual should be zero r=0. Instead of Monte Carlo, Gaussian quadrature is chosen to approximate all the spatial integral terms in the residual formulation, as direct sampling becomes inefficient and inaccurate when there are too many subdomains. Specifically, the displacement field within each mesh element is interpolated by first-order shape functions whose magnitudes are defined by the neural network prediction at the nodal coordinates:


(14) r=[M]a+[R]−[F],

(15) $$\begin{array}{ll} & \left\{ \begin{array}{c} \left[M]=\underset{e}{A}\;[M^{e}]=\underset{e}{A}\;[\sum_{gp}w_{gp}J^{e}[N{]}^{T}]\rho_{0}^{e}[N\right]\\ {}[R]=\underset{e}{A}\;[R^{e}]=\underset{e}{A}\;[\sum_{gp}w_{gp}J^{e}[B{]}^{T}P^{e}],\\ {}[F]=\underset{e}{A}\;[F^{e}]=\underset{e}{A}\;[\sum_{gp}w_{gp}J^{e}[N{]}^{T}]\rho_{0}^{e}b^{e}+p^{e} \end{array}\right. \end{array}$$

where [M], [R], [F] denote the numerical mass, stress divergence, and external force matrices. The symbol $\underset{e}{A}$ denotes the assembly operation for the numerical matrices from their elemental forms to global forms. J=det(F), N, and B denote the Jacobian, shape function, and numerical differentiation operator. All the quantities are interpolated on their corresponding Gaussian points. Besides the weak-formulated residual, the complete training loss will involve initial and boundary conditions, which are detailed in the corresponding Results section.

### Deep learning configuration

PINNs that adopt Monte Carlo approximations for residual computation can be easily parallelized in mini-batches as the loss for each collocation point is calculated independently. On the other hand, residual functions of the proposed weak-formulated PINN are calculated based on shape interpolation at quadrature points within each local mesh element. Therefore, to maximize computation efficiency, all physical quantities are raised to 5th-order tensors. The first three dimensions represent the batch size, number of elements, and the quadrature points, while the last two dimensions represent the original tensor. Pytorch with DeepXDE (58) is used as the physics-informed deep learning library for this study, and all models are trained with the Adam optimizer on an Nvidia RTX 2080 GPU. Other detailed hyperparameters depend on the specific mechanical problem with more details elaborated in the corresponding Result sections.

## Data Availability

All data are included in the main manuscript.
